# Notes and News

**Published:** 1889-03-23

**Authors:** 


### Notes and News.

Collected and Communicated.

We call the attention of our readers to an entertainment which is to be given under the patronage of their Royal Highnesses the Prince and Princess of Wales, the Princess Louise (Marchioness of Lorne), and the Princess Mary, Duchess of Teck, entitled a "May Day Medley," at the Meistersingers' Club, 63, St. James's Street, by permission of the committee, on May 1st and 3rd, in aid of The Hospitals Association. The performances will comprise *tableaux vivants* and a fairy fantasy by children, with music and chorus, by members of the children's orchestra. The whole will be under the management of Mr. Percy Armytage, 1a, Wilton Place, who so successfully organised the Christmas Medley in December last for the benefit of the Mothers' Home, Shadwell, and the National Orphan Home, Ham. We take this opportunity of recognising the good work done by the children's orchestra, through whose instrumentality over £1,000 has been given to various children's charitable institutions in and near London during the past three years.

H.R.H. Prince Albert Victor of Wales has graciously consented to preside at a festival dinner, to be held at the Hotel Metropole on May 29th next, in aid of the funds of the Great Northern Central Hospital, Holloway Road, N., which was opened in July last by their Royal Highnesses the Prince and Princess of Wales.

On March 13th, at the annual general meeting of the governors of the Cancer Hospital, the report, which was adopted, showed that during the past year the work of the hospital had considerably increased. This called for additional support, the reliable income being some £3,000 a year less than the ordinary expenditure.

In Dublin some alarm has been caused by the discovery of a well-defined case of leprosy in the city, which was reported to the Public Health Committee by Dr. Cameron. He was authorised to take whatever measures he deemed necessary to prevent the spread of the terrible malady. Why a single case of leprosy should cause such alarm we do not know. Lepers are not so rare in the United Kingdom as the Dublin Committee seem to imagine; there are generally two or three to be found in London. The measures necessary to prevent the spread of leprosy in this age are very few; and so long as sanitation is considered and general health studied, as it is at present, we need not fear an epidemic of leprosy in England.

To the great regret of the committee of the York County Hospital the following resolution had to be sanctioned at their last meeting: "That the court of trustees be requested to authorise the sale of securities sufficient to realise a sum of £2,000, in order to discharge the present balance against the hospital account, and to meet the cost of the sanitary arrangements required to be provided by the Urban Sanitary Authority."

It is very pleasant to read of the continued prosperity of Suffolk General Hospital, and to see that the committee mean to try and increase their powers of helping the poor. A large sum of money is entrusted to these gentlemen, and it is wise of them not to bury their talent, but rather to seek to expend it to good purpose. The number of in-patients at the hospital increases yearly, and would increase still more if some of the barriers were broken down. A staff of district nurses might also be established with advantage.

The Boscombe Cottage Hospital, which is situated in the centre of a large working population, has an excellent provident dispensary connected with its wards, and numbering nearly a thousand members. The usual Jubilee hospital has sprung up in the neighbourhood, and the Boscombe committee make an earnest appeal that these new brooms will not start a free dispensary, and so discourage the habits of thrift on which Boscombe now prides itself. The request is one which should most certainly be complied with.

On March 11th the annual meeting of the Birmingham and Midland Hospital for Women was held, and Dr. Lawson Tait read the medical report. It stated that the medical board were glad to be able to report favourably of the work of the hospital during the past year. The number of patients admitted to the wards at Sparkhill had been 328; of these, 279 were cases requiring operative treatment. In the out-patient department the number of patients had increased; of the 3,237 applying for relief, 323 were rejected as being unsuitable cases for this hospital.

A Dresden paper tells the following, which is not to the credit of the doctors of Saxony: "A rich man, whose wife was dangerously ill, promised to give the doctor half his fortune if he would only cure the lady. The patient recovered under the doctor's treatment, and the husband sent a large sum of money as the fee. The doctor, however, refused to accept the sum offered, and claimed half the manufacturer's fortune. The latter showed no inclination to part with the moiety of his fortune of 2,000,000 marks or more, and the physician is now seeking satisfaction in a court of law."

The thirty first annual meeting of the Dental Hospital of London was held at the Hospital, Leicester Square, on March 14th, under the presidency of Sir Edwin Saunders. In the report the managing committee congratulated the governors on the continued success and prosperity of the institution; also on the great benefits which the hospital continues to afford to the suffering poor, 51,406 cases having been treated during the year 1888, a large number of them painlessly (under anæsthetics), being 3,965 in excess of those of the previous year. Although, mainly owing to the legacies received during the year, the committee had been able to reduce the mortgage debt on the hospital, there was still a deficit of £3,000 in this account, incurred through the indispensable enlargement of the hospital; and they felt compelled to make a further special appeal for funds to enable them to clear off this encumbrance. The charity is unendowed, and additional funds would enable it to greatly extend its usefulness.

THE following vacancies are announced this week, the amount of salary in each case being given after the name of the institution :—

**Resident Medical Officers.**

North-West London Hospital. Senior Resident Medical Officer.
Bradford Infirmary. Surgeon for Dispensary.—£100, board, etc.
Royal Berks Hospital. Assistant House Surgeon.—Board, etc.
Bethlem Hospital. Two Clinical Assistants.—Board, etc.
Newport Infirmary. House Surgeon.—£100, board, etc.
Stanhope Street Dispensary. Medical Officer.—£105.
Birmingham General Hospital. Assistant House Surgeon.—Board, etc.

**Non-resident Medical Officers.**

Thetford Union. Medical Officer.—£45.
Royal Free Hospital. Assistant Physician.
National Orthopædic Hospital. Registrar and Anæsthetist.—£20.
University College Hospital. Assistant Physician.

---

PRINCE ALBERT VICTOR will open the new buildings of the Harrogate Bath Hospital in July.—Princess Christian will open the new buildings of the Blackheath and Charlton Cottage Hospital in May next.

---

THE Princess Alice Memorial Hospital at Eastbourne was reopened on Saturday last, after an extensive enlargement, the Mayor and the leading residents being present. New wings have been erected by public subscription, but several hundred pounds are needed to cover the extensive outlay.

---

THE steamship *Tainui*, which arrived at Plymouth this week from New Zealand, reports that on calling at Rio de Janeiro she found that yellow fever was raging there with great virulence, the deaths from the disease averaging a hundred per day. There was quite a panic among the inhabitants. Yellow fever broke out on board the *Atrato* during her passage from Rio de Janeiro to Buenos Ayres, and two of her passengers died from the disease. One was an Englishman, a first-class passenger, and the other a third-class passenger.

---

AN earnest appeal for funds comes from Bristol General Hospital, and we specially recommend it to the notice of the many rich families living at Clifton and elsewhere in the neighbourhood. Dr. W. H. C. Newnham, house surgeon, in his report said : "I have again to complain of the want of beds. Daily I turn away deserving cases from our doors, and we are compelled to keep as out-patients many poor people who would get well probably in half the time if they could only be admitted. It is worth noticing that the number of in-patients has been 167 more than in 1887, and 282 more than in 1886. We have seen 1,616 more out-patients than in 1887. Also, we have had 1,154 more casualties than in 1887, or 2,422 more than in 1886. It is an absolute necessity that we should have additional beds." But it is also an absolute necessity that money must be forthcoming to supply and support these extra beds.

---

A NUMBER of workmen at Wallsend are of the opinion that they need an infirmary in that district. There are three large engineering establishments at Wallsend, two shipyards, and various other industries conducive to accidents. Only last week a sailor fell down the hold of a ship and got his head severely cut and bruised. He also broke some of his bones, and had to be taken on an ambulance car to the boat and run to Wallsend. He was then taken up to the town to the doctor, and had his wounds temporarily dressed, after which he was taken back to the boat and run to Newcastle, to be lifted again and taken to the Infirmary, so that for hours this poor fellow was suffering excrutiating pain before having his wounds finally attended to. The above is but an illustration of what frequently occurs, so all will see how necessary it is to have an infirmary in the vicinity, so that cases of accidents can be speedily attended to.

THE Infectious Hospital question at Liverpool does not advance rapidly. The inhabitants of Edge Lane Hall Estate object to a fever hospital in their midst. Considering that the Board pay £600 a-year to the Parkhill Hospital, where at present they have not a single patient, further accommodation does not seem greatly necessary. After two hours and a half of lively discussion last week, the Board could only decide to let the question stand over for a month. Meanwhile the members would do well to find out particulars about the available beds at Netherfield, Grafton, and Parkhill hospitals.

---

THE extension of Aberdeen Infirmary has been begun, and promises to be a big affair. The new building, which is estimated to cost £24,000, will comprise the surgical and part of the clinical and administrative departments of the infirmary, and contains wards for the treatment of those suffering from accidents, from diseases of the eye, etc., in addition to offices for the clerk and treasurer, a board-room, and a dispensary. All the rooms are fourteen feet high from floor to ceiling. Special and careful attention has been bestowed on all the sanitary arrangements of the new hospital. The drainage and ventilation will be carried out on the most approved principles, while the heating of the various rooms will be effected by means of hot-water pipes and large stoves. Round the walls of the wards on the floor-level will be placed perforated horizontal "skirting boxes," through which the cold air from the outside will, after being heated, pass into the wards. The whole of the stairs are of Turin stone, the walls inside plastered with cement, finished with Parian cement, and all the floors will be fireproof. To prevent dust and other dirt accumulating, all angles and corners will be rounded. Bath rooms and other conveniences will be lined with white tiles to a height of six feet, and in the wards enamelled slate dados are proposed to be fixed. An improvement worthy of notice is the proposed erection of balconies on which patients in a convalescent state may take exercise. Snell and Son, of London, and Smith and Kelly, of Aberdeen, are the architects.

---

HERE is another amateur dabbler in drugs carrying away the plums which should fall to the medical profession ! M. Marie de Mayrena, French journalist and officer, was sent to Summatra in 1885 to report on certain pressing matters. Having got through his duties, he wandered on into Further India to the Kingdom of Sédangs. The country struck him as worth opening up, and he got some volunteers from Paris to join him, and then proceeded on his way. They found the first village they came to in a state of epidemic. A sort of jungle fever was playing sad havoc with the natives, and a store of quinine which the travellers had with them not only cured their new hosts, but stamped them as beneficient sorcerers, and secured them the friendship of their patients. M. de Mayrena was elected a chief in recognition of his services, and an escort conducted him into the country. Here he found war progressing. There were several tribes in the country, and they had been fighting each other on and off, after the Chinese method, for many generations. Our hero fought, too, and led his side to victory. Having assured his position, he married the only child of a great chief, and with the assistance of his father-in-law summoned a vast assemblage of chiefs to a national discussion. They met, and he lectured them on the advantages of trade, and the stupidity of fighting ; he gave them the outline of his plans for the formation of a railway and the disposal of their minerals, which were almost unlimited. They listened and departed, to return again, and with one accord to elect him Chief of the Chiefs, or King of the Sédangs. Now, it is very certain M. Marie would never have been a king if it had not been for that quinine ; and we do consider a professional man ought to have scored this conquest !

